# The Management and Diagnosis of Anti-NMDA Receptor Autoimmune Encephalitis in Pregnant Women: A Case Report and Literature Review

**DOI:** 10.3390/medicina59122110

**Published:** 2023-12-01

**Authors:** Alina Potorac, Valentin Nicolae Varlas, Roxana Georgiana Borș, Alexandru Baroș, Monica Cirstoiu

**Affiliations:** 1 Doctoral School, “Carol Davila” University of Medicine and Pharmacy, 050474 Bucharest, Romania; alina.potorac@drd.umfcd.ro (A.P.); roxana-georgiana.bors@drd.umfcd.ro (R.G.B.); 2 Department of Obstetrics and Gynecology, “Carol Davila” University of Medicine and Pharmacy, 050474 Bucharest, Romania; alexandru.baros@umfcd.ro (A.B.); monica.cirstoiu@umfcd.ro (M.C.); 3 Department of Obstetrics and Gynaecology, Filantropia Clinical Hospital, 011132 Bucharest, Romania; 4 Department of Obstetrics and Gynecology, University Emergency Hospital, 050098 Bucharest, Romania

**Keywords:** psychiatric symptoms, cesarean section, gestation, anti-NMDAR autoimmune encephalitis, hallucinations, excellent maternal recovery

## Abstract

*Rationale*: Anti-N-methyl-d-aspartate receptor (NMDAR) encephalitis is a form of autoimmune synaptic encephalitis, often mediated by neuronal surface antibodies. Clinically, it manifests through a diverse range of neurological and psychiatric symptoms, primarily affecting young women with ovarian teratoma, which is rare in pregnant women. *Patient concerns*: We report a case of a 35-year-old multiparous pregnant patient at 38 weeks of gestation presented to the emergency room with seizure, psychiatric symptoms like delirious speech with mystical visual and auditory hallucinations, bradylalia, and retrograde amnesia. *Diagnosis*: The diagnosis of autoimmune encephalitis with anti-NMDA antibodies was concluded by considering the lumbar puncture results, brain imaging, and the patient’s persistent symptoms. *Outcomes*: This case is noteworthy for its rarity and the symptoms’ breadth. At 38 weeks of gestation, the patient underwent a cesarean section, resulting in excellent maternal recovery observed during the 6-month follow-up and good neonatal adaptation. *Lessons*: Our goals include raising awareness about this condition and emphasizing the significance of early diagnosis. This encephalitis is treatable and potentially reversible, underscoring the importance of prompt identification.

## 1. Introduction

Autoimmune encephalitis (AE) includes various groups of autoimmune afflictions that involve the central nervous system (CNS), and the diagnosis is made based on Grau’s criteria [1]. Previous diagnostic criteria of encephalitis typically required an altered mental state and evidence of inflammation. However, AE can deviate from these, with patients lacking consciousness issues, fever, or abnormal cerebrospinal fluid results. Neuroimaging may be normal, and antibody testing results may take weeks. Non-antibody-dependent clinical criteria were developed to aid swift diagnosis and immunotherapy initiation in suspected cases, excluding other potential causes. The diagnostic criteria for anti-NMDAR encephalitis divide cases into two categories, “Probable” and “Definite,” based on specific clinical and laboratory parameters. In the “Probable” category, a rapid onset (within 3 months) of at least four major symptom groups, including abnormal behavior, speech dysfunction, movement disorder, decreased consciousness, and autonomic dysfunction, is required.

Additionally, abnormal laboratory test results and the reasonable exclusion of other disorders are necessary. Diagnosis in this category is also possible if three symptom groups are present along with a systemic teratoma. In the “Definite” category, the presence of one or more major symptom groups, along with IgG anti-GluN1 antibodies, confirms the diagnosis after reasonably excluding other disorders. Confirmation tests, including live immunohistochemistry or cell-based assays, should be conducted on neurons or tissue, particularly when only serum is available. AE is often a paraneoplastic syndrome associated with ovarian, testicular, lung, breast, and Hodgkin’s lymphoma [2].

Autoimmune encephalitis with N-methyl-D-aspartate (NMDA) receptors is a seldom-seen yet severe disorder affecting the CNS. It triggers inflammation within the brain and spinal cord, brought on by an abnormal immune response where antibodies target NMDA receptors, the N1 subunit, and crucial proteins for nerve cell functionality, with a variety of major neurological symptoms, like memory loss, dyskinesias, dysautonomia, confusion, hallucinations, seizures, central hypoventilation, and decreased level of consciousness [3]. Managing autoimmune encephalitis with NMDA receptors during pregnancy is intricate, necessitating a delicate balance between the mother’s treatment and the safety of the fetus. Anti-NMDAR autoimmune encephalitis is a severe affliction but treatable, with more than 60% of cases in young women. Numerous studies have indicated complications in pregnant patients, such as dysautonomia, seizures, or central hypoventilation. Additionally, evidence suggests that the transplacental transfer of NMDAR antibodies may lead to acute encephalopathy, or even prove fatal for newborns, potentially contributing to the development of autistic spectrum disorders in the long term [4,5]. This condition was described for the first time in 2005 in young patients with ovarian teratoma [6]. 

When addressing this condition, the healthcare provider must carefully consider the potential harmful effects of treatments on the fetus, weighing them against the benefits for the mother. This balance is particularly crucial during the first trimester of pregnancy. The administration of anti-seizure medications (such as carbamazepine and phenytoin), immunomodulatory drugs (such as cyclophosphamide), or performing radiological assessments to identify underlying neoplasia (such as abdominal and pelvic computed tomography with contrast enhancement for ovarian teratoma) during this period is associated with an elevated risk of congenital malformations, such as spina bifida and cardiac anomalies, as well as newborn distress [7].

In the following case study, we present the situation of a young woman diagnosed with anti-NMDAR encephalitis during the third trimester of pregnancy. Our objectives are to emphasize the positive outcomes for the mother, and raise awareness about this condition and the significance of early diagnosis. This encephalitis is treatable and potentially reversible, underscoring the importance of prompt identification. Additionally, we aim to educate gynecologists, psychiatrists, anesthetists, and neurologists about this potential cause of psychiatric and neurological symptoms during pregnancy. 

## 2. Case Report

A 35-year-old multiparous pregnant patient at 38 weeks of gestation presented to the emergency room following a spontaneous fall from the bed while she was admitted to another medical facility, experiencing a loss of consciousness for 10 min and lower limb myoclonus, followed by psychomotor agitation, delirious speech with mystical visual and auditory hallucinations, bradylalia, and retrograde amnesia. There were no prodromal symptoms of encephalitis. Her medical history shows that the patient has genetic thrombophilia (heterozygous variants of MTHFR). Regarding hereditary antecedents, there are no known autoimmune diseases.

**Figure 1:**
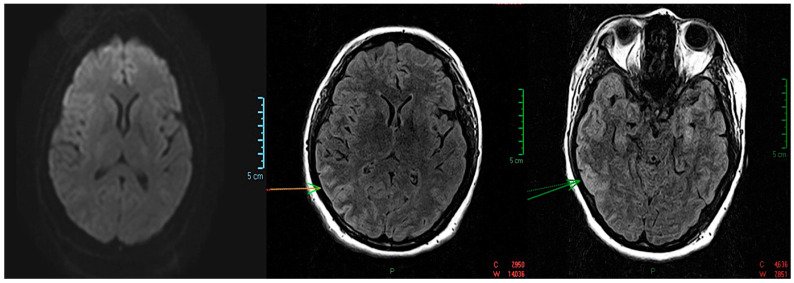
The initial native brain MRI revealed: axial hyperintensity on the T2 and FLAIR sequences of the right temporo-occipital cortex; a “Swollen” appearance of the right temporo-occipital cortex, in hyper signal T2 and FLAIR (arrow), without a correspondent on the diffusion or T2* sequences, associated with moderate erasure of the intergyral grooves but without a mass effect on the midline structures; the imaging appearance raises the suspicion of a temporal cortical edema—parietal; no other abnormalities of brain signal or morphology, and no lesions with diffusion restriction or endocranial bleeding scars; normally positioned and sized ventricular system.

**Figure 2:**
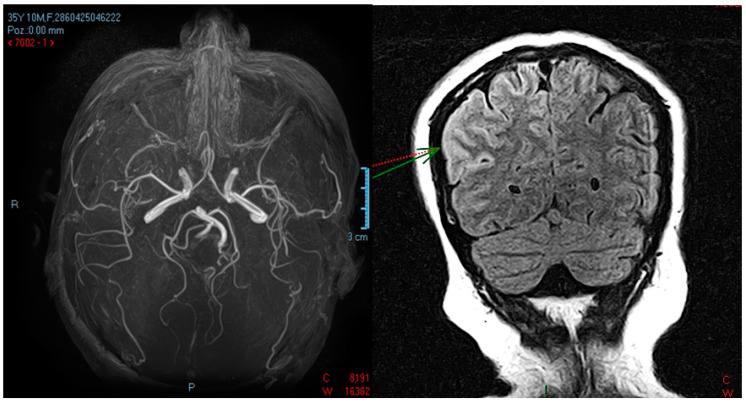
Three-dimensional Time-of-Flight (TOF) MR angiography: main endocranial arteries with fast flow and normal morphology. Asymmetry on the coronal FLAIR sequence (arrow).

After admission, a neurology consultation was performed. At the time of the consultation, the patient did not exhibit signs of meningeal irritation, had preserved oculomotor function without nystagmus, and showed no coordination or sensory disturbances. She had slow speech (bradylalia) and experienced episodes of visual and auditory hallucinations with a mystical character. A psychiatric consultation was also conducted, raising the suspicion of schizophrenia. As a result, treatment with carbamazepine 400 mg/day, lorazepam 2 mg/day, diazepam, and haloperidol 50 mg/day was initiated. An emergency native cerebral CT was performed, yielding normal results, followed by a cerebral MRI that identified changes in the temporo-occipital cortex, with more pronounced asymmetry on the coronal FLAIR sequence. It also showed water restriction on the diffusion-weighted sequence, indicating a non-specific MRI appearance, possibly in the context of recent diffuse axonal injuries. On the native cerebral MRI, a “swollen” appearance of the right temporo-occipital cortex was observed, showing hyperintensity on the T2 and FLAIR sequences without corresponding findings on the diffusion-weighted or T2 sequences. This was associated with the moderate effacement of the intergyral sulci, raising the suspicion of temporo-parietal cortical edema. Three-dimensional Time-of-Flight (TOF) MR angiography revealed the main intracranial arteries to have rapid flow, normal morphology, and a normally positioned and sized ventricular system (Figure 1 and Figure 2).

On the same day, after admission to the Obstetrics–Gynecology Department, the decision was made to perform a cesarean section to enable the initiation of psychiatric treatment contraindicated during pregnancy and to continue further imaging investigations for establishing the diagnosis and planning subsequent management. The newborn weighed 3050 g and suffered transient respiratory distress, with Apgar 8 at 1 min and 9 at 5 min. Postoperatively, the patient was admitted for two days to the intensive care unit for monitoring and advanced support. 

After being transferred back to the Obstetrics–Gynecology Department, considering the symptoms, treatment with carbamazepine 400 mg/day, lorazepam 2 mg/day, diazepam, and haloperidol 50 mg/day was initiated, without any response. Following a subsequent neurological consultation, the suspicion of viral encephalitis arose, and empirical treatment was initiated with acyclovir 1000 mg iv every 8 h, ceftriaxone 2 g iv every 12 h, and methylprednisolone 1 g/day iv; the psychiatric treatment continued with olanzapine, diazepam, carbamazepine, and haloperidol, as needed. The long-term video electrocardiogram (video-EEG) provided a recording without epileptic discharges, generalized slowing activity, or delta waves.

A lumbar puncture was performed on the fourth postoperative day. Cerebrospinal fluid (CSF) analysis revealed 29 white cells per mm^3^ (85.3% lymphocytes), 49 mg/dL glucose, 32.52 mg/dL proteins, positive oligoclonal bands, antineuronal antibodies, and anti-Ri weakly positive and positive antibodies against NMDA glutamate receptors (Table 1), with no organisms on the Gram stain, negative CSF cultures, and, on the Ziehl–Neelsen stain, no acid-alcohol-resistant bacilli were detected.

Following the cerebrospinal fluid analysis and the positive results, due to the risk of a paraneoplastic neurologic syndrome, a thoracic-abdominal-pelvic CT scan with contrast was performed. The results revealed images of thrombi partially occupying the distal portion of the right pulmonary artery, with extension into the lower lobe, and a quasi-occlusive thrombosis involving the left common iliac vein, the external iliac vein up to the level of the left femoral vein and deep inguinal vein, without the presence of a teratoma or lung cancer. 

Considering the results of the lumbar puncture, brain imaging, and the patient’s persistent symptoms, the diagnosis of autoimmune encephalitis with anti-NMDA antibodies was concluded. Treatment continued with methylprednisolone without a favorable response, followed by one course of cyclophosphamide without clinical improvement. Subsequently, one course of Rituximab was administered, with a progressive improvement in neurological symptoms, enhancement of delirious episodes, and no neurological signs of focal deficits. The IV Ig treatment was not initiated due to the lack of resources at that time.

The second native brain MRI and IV post-contrast were performed 12 days after the first MRI, and, when compared with the previous examination, they highlighted the following:

- a slight regression in the size and intensity of the T2 and FLAIR hyper signal, without restrictions on the diffusion sequence, not capturing the right temporo-occipital, associating a “swollen” aspect of the cortex with a slight erasure of the intergyral grooves, but without a mass effect on median line structures;
- small non-specific demyelinating lesions in hyper signal T2 and FLAIR, 2–4 mm, without diffusion sequence restriction, non-capturing, located in the frontoparietal subcortical and bilateral periventricular;
- no restrictions on the diffusion sequence, no hemorrhagic stigmata visible on the T2* sequence, no pathological intra/extra neuraxial contrast uptake or at the leptomeningeal level;
- normally positioned and sized ventricular system.

At the 6-month follow-up, the patient showed no pathological changes in paraclinical examinations, and a thoracic-abdominal-pelvic CT scan with contrast was performed, excluding any neoplastic pathology at that moment associated with autoimmune encephalitis. The permeabilization of the pulmonary artery trunks was also noted. It was decided to stop immunosuppressive therapy due to the favorable progression and complete resolution of symptoms, and to continue the follow-up, considering the possibility of an occult or a developing neoplasm.

## 3. Discussion

Anti-NMDAR encephalitis is an autoimmune condition characterized by organ and tissue damage caused by antibodies or T cells; few cases are described in the literature (Table 2). Currently, although the precise underlying pathogenesis of this disorder remains incompletely elucidated, an increasing body of evidence suggests that antibodies developed in response to various potential triggers (such as tumors or infections) likely cross-react with synaptic proteins, with the NMDAR being the most commonly affected [8]. Research indicates that its origins may be linked to the immune regulation of sex hormones [9], genetic factors from X and Y chromosomes, specific immune responses related to pregnancy, and a combination of genetic and environmental influences [10]. 

Estrogen and progesterone levels fluctuate throughout pregnancy, with estrogen stimulating B cell survival and antibody production while inhibiting T cell expansion, potentially exacerbating autoimmune disorders. The intricate immune response of a mother to the fetus, perceived as a “half-self, half-non-self” antigen, increases the susceptibility to autoimmune diseases [8,11]. In the case presented, from the clinical and paraclinical investigations, and the complete resolution of symptoms at the 6-month follow-up, it was concluded that it was not a paraneoplastic syndrome but rather an immune response related to pregnancy and external factors.

The underlying causes of anti-NMDAR encephalitis during pregnancy have not been fully elucidated. A characteristic of this condition is its higher prevalence among women of reproductive age, often coinciding with ovarian teratomas. We can speculate that there might be a connection between pregnant patients predisposed to anti-NMDAR encephalitis and the presence of ovarian teratomas [12,13,14]. In our case, after confirming the diagnosis, transvaginal and transabdominal ultrasounds and abdominal CT scans (after the cesarean section delivery) were performed, revealing the absence of any teratoma. Furthermore, only 10 out of 30 (33.3%) cases studied in the literature presented a teratoma.

Lim et al., in their study conducted in Korea, found that the predominant symptoms among patients were psychiatric (68.8%), with epileptic seizures following closely at 50.0% [15]. In our case, the patient experienced a single epileptic seizure at the onset of the disease. In the literature review, only 36.6% of patients presented seizures (Table 2).

**Table 2:** A literature review of cases of anti-NMDAR autoimmune encephalitis during gestation.

Author/ Year	Age	Presenting Symptoms	GA (wks)	Teratoma	Treatment	History of Autoimmune Disorders	Imaging to Exclude Teratoma	Mother’s Outcome	Perinatal Outcomes	Follow-Up
Kumar Ref. [16], 2010	19	Headache followed by behavior abnormalities	14	Present	IV Ig, IV methyl-prednisolone, resection of teratoma	NS	MRI—left teratoma	Normal	Normal	2 m
Kumar Ref. [16], 2010	20	Behavior abnormalities	8	Absent	IV Ig	Bilateral teratoma removed at 16 y	CT (bilateral teratoma)	Minimal deficits	Aborted	NS
Kumar Ref. [16], 2010	19	Behavior abnormalities	17	Absent	IV methylprednisolone	NS	MRI/US	Normal	Normal	NS
Ito Ref. [17], 2010	19	Dyskinesia, behavior abnormalities	17	Absent	Corticosteroids	No history	MRI/US	Normal	Normal	NS
McCarthy Ref. [18], 2012	32	Behavior abnormalities, autonomic symptoms	8	Present	IV methylprednisolone, plasmapheresis, resection of teratoma	NS	MRI/US-negative for teratoma; left teratoma at C-section	Normal	Normal	NS
Jagota Ref. [19], 2014	18	Fever, orolingual movements, eye deviation	9	Absent	Azathioprine, IV Ig	NS	MRI/CT	The patient died due to sepsis.	Baby survived, delivered at 34 wks (NVD)	Baby follow-up 3 y; the patient died
Shanani Ref. [20], 2015	26	Behavioral abnormalities and headache	22	Absent	Oral corticosteroids, IV methylprednisolone, plasmapheresis	NS	US	Normal	Normal	18 m
Kim Ref. [3], 2015	28	Abnormal behavior, an epileptic seizure, hypoventilation, and dyskinesia	7	Present	IV Ig, IV methyl-prednisolone; oral corticosteroids, plasmapheresis, resection of teratoma	NS	US negative, CT—right ovarian teratoma	Slight cognitive function deficits	Aborted	12 m
Mathis Ref. [21], 2015	21	Behavior abnormalities	10	Absent	IV Ig, IV methyl-prednisolone	NS	MRI	Slight memory impairment	Normal	9 m
Chan Ref. [22], 2015	23	Hallucinations, confusion, fever, disinhibited behavior	1st trimester	Present	IV methylprednisolone, plasmapheresis, Rituximab, resection of teratoma	NS	CT—right ovarian teratoma	Normal	Aborted	18 m
Lamale-Smith Ref. [23], 2015	24	Confused, catatonia, disoriented	20	Absent	IV methylprednisolone IV Ig	NS	Imaging (NS)—no teratoma	Disinhibition, memory impairment	Normal	12 m
Xiao Ref. [24], 2017	24	Visual and auditory hallucinations	28	Absent	IV Ig, IV methyl-prednisolone, bilateral ovarian wedge resection	NS	MRI	Normal	Normal	12 m
Keskin Ref. [25], 2019	27	Visual hallucination, headache, seizure	18	Absent	IV Ig, IV methyl-prednisolone, plasmapheresis	NS	US	Death	Fetal demise	Patient and baby died
Tailland Ref. [26], 2020	37	Orofacial dyskinesia, pyramidal bilateral syndrome	18	Absent	IV Ig, IV methyl-prednisolone	NS	MRI	Left hemiparesis, cognitive impairment	Normal	NS
Jung Ref. [27], 2020	28	Focal seizure, depression, headache	24	Absent	IV Ig, IV methyl-prednisolone, oral corticosteroids, Rituximab	NS	US/MRI	Normal	Normal	24 m
Joubert Ref. [4], 2020	23	Nausea, visual hallucination, delirium	8	Present	IV Ig, resection of teratoma	NS	NS	Normal	Normal	NS
Joubert Ref. [4], 2020	20	Motor aphasia, behavior abnormalities, dysarthria	12	Absent	IV Ig	NS	NS	Normal	Normal	NS
Joubert Ref. [4], 2020	25	Behavior abnormalities and epilepsy	5	Absent	IV Ig, plasmapheresis, Rituximab	NS	NS	Poor responder	Normal	NS
Joubert Ref. [4], 2020	31	Cognitive fluctuation, orofacial dyskinesia, delirium, memory deficits	20	Present	IV Ig, Rituximab, cyclophosphamide, resection of teratoma	NS	NS	Poor responder	Normal	NS
Joubert Ref. [4], 2020	37	Bulbar palsy and hemifacial sensitivity deficit	33	Absent	IV Ig, cyclophosphamide	NS	NS	Normal	Normal	NS
Joubert Ref. [4], 2020	19	Delirium, visual agitation, hallucination	25	Present	IV Ig, cyclophosphamide, resection of teratoma	NS	NS	Poor responder	Normal	NS
Sperling Ref. [28], 2021	34	Seizures, uncontrolled laughing, emotional lability, short-term memory loss, head banging against the wall, insomnia	14	Present	IV Ig, IV methyl-prednisolone, plasmapheresis Rituximab, levetiracetam, Fosphenytoin, Valproate, resection of teratoma	NS	MRI—right teratoma	Poor memory	Normal	6 m
Boniface Ref. [29], 2021	22	Hallucinations, nose-bleeds, nausea, vomiting, movements of the extremities, seizures	23	Present	Propofol, levetiracetam, acyclovir, ampicillin, vancomycin, ceftriaxone, resection of teratoma, plasmapheresis	NS	US—right teratoma	Significant automatisms (orofacial and hand-jerking dyskinesias)	Normal	1 m
Liu Ref. [8], 2021 (same case) Third pregnancy Fourth pregnancy	19	Lowered consciousness, restlessness, involuntary movement of the oral and facial muscles, and high muscle tension in the limbs	8	Absent	IV Ig, IV methyl-prednisolone, Rituximab	NS	US	Normal	Aborted	12 m
	21	Similar psychiatric and epileptic symptoms	10	Absent	IV Ig, IV methyl-prednisolone, levetiracetam, olanzapine	NMDA encephalitis in the previous pregnancy	US	Poor memory and mild cognitive impairment	Aborted	3 m
Reisz Ref. [30], 2022	31	Bizarre demeanor, alteration of cognitive status, mutism, catatonic-like status, epileptic seizures, choreoathetosis, autonomic disorders	17	Absent	Plasmapheresis	NS	MRI	Spastic paraparesis, peculiar childlike voice, immature behavior	Aborted	24 m
Dono Ref. [31], 2022	29	Continuous, ongoing, focal motor seizures involving the right side of the face, emotional liability with sudden changes in mood and behavior, sialorrhea	7	Absent	Lacosamide, prednisone, prednisolone, PLEX	NS	MRI/US	Normal	Normal	3 m
Fredrich Ref. [32], 2022	19	Seizures, psychotic behaviors, global hyperreflexia, left Babinski, continuous right upper extremity twitching, and oral automatisms	11	Absent	Lacosamide, lamotrigine, levetiracetam, clonazepam, IV Ig, IV Solumedrol, PLEX cyclophosphamide Rituximab	NS	NS	Fully ambulatory with mild cognitive deficits	Normal	36 m
Scorrano Ref. [33], 2022	29	Tonic–clonic seizure, with minor head trauma and subacute onset of psychiatric symptoms	6	Absent	Prednisone, plasmapheresis, levetiracetam, lacosamide	NS	MRI	NS	Low birth weight, respiratory distress, spina bifida	NS
Bansal Ref. [34], 2023	36	Aggressive, delirious, minimally directable, and with pressurized speech, impairment of insight and judgment, combative, hallucinating	17	Present	Lorazepam, diphenhydramin, haloperidol, ketamine, Risperidone, IV Ig, resection of teratoma	NS	US—right teratoma	Normal	Normal	1 m
Our case	35	Spontaneous fall, loss of consciousness for 10 min, lower limb myoclonus, retrograde amnesia, psychomotor agitation, delirious speech, mystical visual/ auditory hallucinations, bradylalia	38	Absent	Acyclovir, IV ceftriaxone, IV methylprednisolone, olanzapine/carbamazepine, Rituximab	NS	US/CT	Normal	Normal	6 m

GA—gestational age; IV Ig—intravenous immunoglobulin; IV—intravenous; PLEX—plasma exchange; min—minutes; wks—weeks; m—months; y—years, NS—non-specified.

The average age of the studied patients was 25.6 years (range 19–37), and the average gestational age was 15.5 weeks (range 5–33). According to the distribution by trimesters, the condition was encountered in the first trimester of pregnancy in 16 cases (53.33%), in the second trimester in 12 cases (40%), and in 2 cases in the third trimester (6.67%) at 28 weeks and 33 weeks. The case we presented is the only one diagnosed at term at 38 weeks.

In the case of most pregnant patients, immunomodulatory therapies include those recognized as primary treatment options, such as steroids, intravenous immunoglobulin (IV Ig), and plasma exchange. The findings reported in the existing literature affirm the generally well-tolerated nature of these treatments during pregnancy. Additionally, Rituximab, often considered a secondary line of treatment, has been limited to a small patient population but has shown favorable tolerance levels. Previous retrospective analyses and literature reviews that focused on pregnant individuals with autoimmune demyelinating disorders treated with Rituximab have not revealed any adverse effects on the patients or their newborns [35]. Immunomodulatory treatment involving systemic corticosteroids appears to be safe during pregnancy. Pregnant women undergoing corticosteroid therapy typically exhibit a minimal risk of experiencing significant congenital malformations. However, there is a possibility of premature rupture of amniotic membranes and the birth of low-weight babies [36]. 

Intravenous immunoglobulins are regarded as safe during pregnancy. Numerous reports in the obstetric literature detail the use of IV Ig therapy to address various conditions encountered during pregnancy [37]. Additionally, several case reports and series have demonstrated positive outcomes in pregnant patients with autoimmune epilepsies (AEs) treated with IV Ig, with a low incidence of side effects and infectious complications [31]. In our case, the treatment with corticosteroids and Rituximab was initiated after the cesarean section delivery, and plasma exchange (PLEX) was not required. The clinical response was positive, and the 6-month follow-up revealed a complete resolution of the symptoms (Table 2). 

The lack of guidelines and the rare incidence of this condition explain the uneven therapeutic trials. From the analysis of the medication administered, it was observed that 21 cases were treated with IV Ig, 17 cases with IV methylprednisolone, 10 cases with plasmapheresis, and 4 cases with cyclophosphamide. As a result, dual IV Ig therapy, especially with IV methyl prednisolone, is the most effective.

The maternal prognosis was favorable in 14 cases (48.2%), with minimal/mild cognitive deficits in 4 cases (13.8%), and unfavorable / poor responders in 9 cases (31%). Two deaths (6.9%) were recorded. This information leads to the conclusion that anti-NMDA receptor autoimmune encephalitis continues to increase morbidity and mortality, and represents an element of differential diagnosis in medical practice.

The fetal prognosis was favorable in 22 (73.33%) cases, with abortions in 7 (23.33%) cases, and one death of the child at the age of 3 years. Regarding the follow-up, data were reported in 16 cases, the average being 13.47 months (1–36 months), which implies the need for the long-term monitoring of these children.

The limitation of this study is the search in a single database of cases with this condition. From the analysis of the studied cases, it can be seen that the diagnosis is difficult to achieve at the time of admission, requiring a series of additional laboratory and imaging analyses during which the treatment is non-specific. The strength of this study is represented by the rarity of the appearance of this pathology in the third trimester of pregnancy, more precisely, at term, because the vast majority of these cases are reported in the literature predominantly in the first two trimesters, which can make it difficult to establish the diagnosis and delays the initiation of treatment with repercussions on maternal prognosis.

To better decipher this pathology that occurs mainly in the first two trimesters of pregnancy, in 93.1% of the cases found in the literature, a multi-omics approach could be essential in understanding the etiology of this condition, the heterogeneous manifestation of the phenotype in the case of sexual chromosomal abnormalities, and for the development of therapeutic management adapted to each case.

## 4. Conclusions

Autoimmune encephalitis with NMDA receptors during pregnancy presents a complex medical challenge. Pregnancy serves as an inducer for anti-NMDAR encephalitis; moreover, the exclusion of a paraneoplastic syndrome is imperative, and also the rigorous follow-up, considering the possibility of an occult or a developing neoplasm. Early detection and suitable treatment, coupled with comprehensive medical and emotional support, significantly impact the lives of affected patients and their newborns. Collaboration among medical professionals is crucial in formulating a treatment plan that mitigates risks for both individuals.

## Figures and Tables

**Table 1:** Cerebrospinal fluid analysis.

Test	Result	Normal Range/Units
RBC	367	cells/mm^3^
TNC	29	cells/mm^3^
- Mononuclear cells %	85.3	%
- Polymorphonuclear cells %	14.7	%
- Mononuclear cells	19	cells/mm^3^
- Polymorphonuclear cells	3	cells/mm^3^
Glucose	49	40–70/mg/dL
Antineuronal antibodies		
- Anti-Yo (PCA1)	negative	negative
- Anti-Hu (ANNA1)	negative	negative
- Anti-recoverin	weakly positive	negative
- Anti-SOX1	negative	negative
- Anti-Titin	negative	negative
- Anti NMDAR	positive	negative
HSV-1	negative	negative
HSV-2	negative	negative

## Data Availability

The data can be obtained from the corresponding author upon reasonable request.
